# Decoding the Cornea-Glaucoma Association: Evidence From Mendelian Randomization

**DOI:** 10.1167/iovs.66.9.22

**Published:** 2025-07-08

**Authors:** Victor A. de Vries, Anita Szabo, Joëlle E. Vergroesen, Siyin Liu, Karin A. van Garderen, Kirithika Muthusamy, Petra Liskova, Lubica Dudakova, Anthony P. Khawaja, Stephen J. Tuft, Caroline C. W. Klaver, Alice E. Davidson, Wishal D. Ramdas

**Affiliations:** 1 Department of Ophthalmology, Erasmus Medical Center, Rotterdam, The Netherlands; 2 Department of Epidemiology, Erasmus Medical Center, Rotterdam, The Netherlands; 3 UCL Institute of Ophthalmology, London, United Kingdom; 4 Moorfields Eye Hospital, London, United Kingdom; 5 First Faculty of Medicine, Charles University and General University Hospital in Prague, Prague, Czech Republic; 6 NIHR Biomedical Research Centre, Moorfields Eye Hospital NHS Foundation Trust & UCL Institute of Ophthalmology, London, United Kingdom; 7 Department of Ophthalmology, Radboud University Medical Center, Nijmegen, The Netherlands; 8 Institute of Molecular and Clinical Ophthalmology, University of Basel, Basel, Switzerland

**Keywords:** open-angle glaucoma (OAG), intraocular pressure (IOP), central corneal thickness (CCT), genetic risk score (GRS)

## Abstract

**Purpose:**

We combined classical association analyses with one-sample and two-sample Mendelian randomization (MR), to comprehensively assess the causal relation among central corneal thickness (CCT), corneal hysteresis, Fuchs endothelial corneal dystrophy (FECD), and open-angle glaucoma (OAG).

**Methods:**

We analyzed data from a large population-based cohort study (the Rotterdam Study), an FECD case-control study, and genome wide association study summary statistics. We defined OAG as reproducible visual field loss, independent of IOP. Multivariable regression was performed. One-sample MR was performed using the same regression models, with the corresponding genetic risk score (GRS) as independent variable. Two-sample MR was performed using inverse variance weighted, MR Egger, weighted median, simple mode, and weighted mode methods.

**Results:**

In total, 303 participants with OAG and 10,598 controls from the Rotterdam Study were included, with 753 FECD cases from the FECD cohort. The odds ratio (OR) 95% confidence interval (CI) of OAG was 0.67 (95% CI = 0.56–0.81) per standard deviation (SD) increase in CCT (*P* < 0.001). However, one-sample MR showed no significant association between a CCT-GRS and OAG (*P* = 0.688). Two-sample MR found an OR (95% CI) of 1.23 (95% CI = 1.06–1.42) for each SD increase in the CCT instrumental variable. We observed no association between an FECD-GRS and OAG (*P* = 0.946).

**Conclusions:**

We found no evidence for a causal link between CCT and OAG. Nevertheless, CCT measurements are still valuable for population-based risk stratification. We found no clear relationship between FECD and OAG.

Glaucoma is the primary cause of irreversible blindness globally, affecting approximately 1% to 3% of individuals aged 40+ years worldwide.^1^ Primary open-angle glaucoma (OAG) is insidious in its onset, as symptoms often only arise in advanced stages. Therefore, early screening initiatives are crucial. Identifying risk factors for OAG is indispensable for optimizing screening initiatives and increasing our understanding of its etiology. Established risk factors are older age, higher intraocular pressure (IOP), and a positive family history of OAG.^2^ Several other risk factors remain disputed, most notably central corneal thickness (CCT).

The Ocular Hypertension Treatment Study reported an increased conversion from ocular hypertension to OAG in patients with a thinner CCT.^3^ However, an inclusion criterion for this study was a measured IOP of 24 to 32 millimeters of mercury (mm Hg) in one eye (with 21–32 mm Hg in the fellow eye). An increased CCT can bias IOP measurements toward a higher measured IOP compared to the true pressure in the eye. Thus, participants with a thicker CCT might have been recruited despite having a relatively low true IOP, and therefore a lower risk for OAG. Since then, the Los Angeles Latino Eye Study and the Early Manifest Glaucoma Trial have both reported an association between thinner CCT and increased OAG risk.^4^^,^^5^ However, in both studies, this association was observed when IOP was included as a covariate in the model. Simulation studies have demonstrated that adjusting for IOP in this context can introduce the risk for collider bias, potentially generating spurious associations where none exist.^6^ This ambiguity can have substantial clinical impact, because without a clear understanding of the relation between CCT and OAG it is difficult to develop clear guidelines on CCT interpretation for OAG screening. This may lead to unnecessary referrals for patients with high CCT and missed OAG diagnosis or treatment delays for those with low CCT.

Similar to CCT, the relation between Fuchs endothelial corneal dystrophy (FECD) and OAG has been disputed.^7^^–^^11^ FECD is a corneal disorder characterized by loss of endothelial cells and small excrescences of Descemet membrane. For both, OAG and FECD, a common proposed mechanism is oxidative stress. Unfortunately, current reports are still limited by the relatively low prevalence of both OAG and FECD (1%–3% and 6%–7%, respectively^1^^,^^12^), and the risk of confounding bias from shared lifestyle/environmental risk factors.

In this study, we leveraged data from a large population-based cohort study, an FECD case-control study, and genome wide association studies (GWAS) summary statistics, to address these knowledge gaps. We utilized classical association analyses, one-sample, and two-sample Mendelian randomization (MR) experiments, to generate a holistic assessment of the true relation among CCT, IOP, and OAG.

## Methods

**Figure 1.**
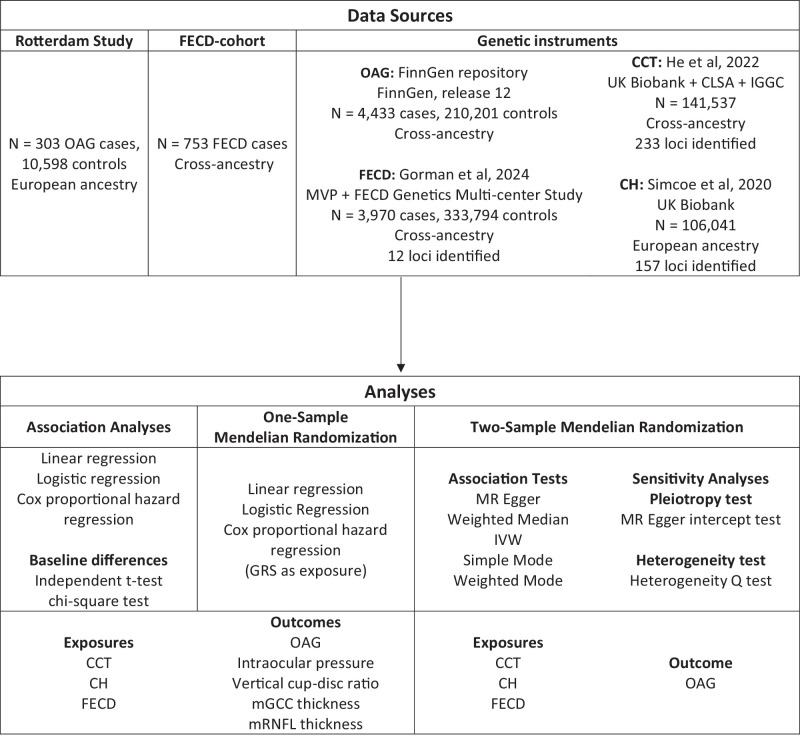
Study design overview. CCT, central corneal thickness; CH, corneal hysteresis; CLSA, Canadian Longitudinal Study on Aging; FECD, Fuchs endothelial corneal dystrophy; IGGC, International Glaucoma Genetics Consortium; IVW, inverse variance weighted; MVP, Million Veterans Program; mGCC, macular ganglion cell complex; MR, Mendelian randomization; mRNFL, macular retinal nerve fiber layer; OAG, open-angle glaucoma; UK, United Kingdom.

This study followed the STROBE-MR guideline for reporting MR results.^13^ An overview of data sources and analyses is presented in Figure 1.

### Ethics Statement

The Medical Ethics Committee of Erasmus MC (registration number MEC 02.1015) and the Dutch Ministry of Health, Welfare, and Sport (Population Screening Act WBO, license number 1071272-159521-PG) approved the use of the individual-level data in the Rotterdam Study (RS) participants. The RS personal registration data collection is filed with the Erasmus MC Data Protection Officer under registration number EMC1712001. The RS is entered into the Netherlands National Trial Register (NTR; www.onderzoekmetmensen.nl) and the World Health Organization (WHO) International Clinical Trials Registry Platform (ICTRP; www.who.int/ictrp/network/primary/en/) under shared catalog number NL6645/NTR6831. The FECD patient recruitment was approved by the Research Ethics Committees of University College London (UCL; 22/EE/0090), Moorfields Eye Hospital London (13/LO/1084), and the General University Hospital Prague (151/11 S-IV). All participants provided written informed consent following the Declaration of Helsinki.

### Rotterdam Study

The main association analyses were performed in the RS, a prospective population-based cohort study of people living in Ommoord, a district of the city of Rotterdam.^14^ The RS consists of four cohorts, of which the first three (RS-I, RS-II, and RS-III) were used for this study. Inclusion of these 3 cohorts started in 1991, 2000, and 2006, respectively. Participants were examined every 4 to 5 years following a standardized protocol, which included visual field testing at baseline and during follow-up for the presence of OAG. Participants underwent an extensive physical examination at the research center as well as a home interview including questionnaires on lifestyle factors and medical history. Details have been published previously.^15^ In short, visual field loss was considered glaucomatous if no other cause could be identified, and no homonymous defects or recognizable patterns like rim artifacts were observed. Hospital records were retrieved for all participants with visual field loss. Participants with other possible causes of visual field loss, signs of anterior chamber angle-closure, secondary glaucoma, pseudoexfoliation, or pigment dispersion syndrome were excluded. OAG was defined as glaucomatous visual field loss in at least one eye, independent of IOP. The IOP was measured using Goldmann applanation tonometry (Haag-Streit AG, Bern, Switzerland), except for the last visit of RS-I (RS-I-7), which was measured using a Topcon TRK-2P non-contact tonometer (Topcon Corporation, Tokyo, Japan). If the participant used IOP-lowering medication, the measured IOP was divided by 0.7 to estimate the untreated IOP, if the participants had undergone IOP-lowering surgery, the IOP was set to 30 mm Hg.^16^^,^^17^ Data on usage of IOP-lowering medications (including anatomic therapeutic chemical codes) was obtained from 7 fully automated pharmacies using a centralized computer network in the study district from January 1, 1991, onward. Vertical cup-disc ratio (vCDR) measurements were extracted from simultaneous stereo color photographs of the optic nerve head (Topcon ImageNet System; ImageNet, Topcon Corporation, Tokyo, Japan), or the Heidelberg Retina Tomograph (HRT; Heidelberg Engineering, Dossenheim, Germany) was used to calculate the linear cup-disc ratio (CDR), which was used as an equivalent outcome for the vCDR analyses (N with linear CDR = 3615, 33.2% of the total cohort).^18^ Optical coherence tomography (OCT) scans were obtained from September 2007 onward, using initially the Topcon 3D-OCT 1000 mk2, since August 2011 the Topcon 3D-OCT 2000, and finally since January 2022 the Topcon DRI Triton OCT (Topcon, Tokyo, Japan). Using an in-house deep learning model that relies on a convolutional neural network, we calculated the average thickness of the macular retinal nerve fiber layer (mRNFL) and macular ganglion cell complex (mGCC) over the surface area of the Early Treatment Diabetic Retinopathy Study (ETDRS) grid, excluding the central subfield (i.e. the fovea). For OAG cases, we used IOP, vCDR, and OCT measurements of the affected eye. If both eyes were affected or unaffected, a random eye was selected. DNA extraction was performed using whole blood samples following standardized and previously described protocols.^14^ Genotyping was performed using both the Infinium II HumanHap550(-Duo) (RS-I and RS-II) and 610-Quad Genotyping BeadChip (RS-I and RS-III; Illumina, San Diego, CA, USA). Imputation of markers was performed using the TOPMed reference panel. We excluded variants with single nucleotide polymorphism (SNP) call rate <0.95, minor allele frequency <0.01, Hardy–Weinberg equilibrium *P* value < 1.0*10^−7^, or MachR^2^ <0.3.

### FECD Cohort

We combined the RS data with data from a multi-center FECD cohort. This cohort served two purposes. First, to validate the FECD genetic risk score (GRS), ensuring that the analyses in the RS using the FECD-GRS were reliable. Second, to assess if the FECD cases had any differences in IOP compared with the controls. The FECD cohort was recruited from September 2009 to July 2023 from Moorfield Eye Hospital in London, United Kingdom, and the General University Hospital in Prague, Czech Republic.^19^ All participants were diagnosed with FECD based on the documented finding of confluent corneal guttae seen by slit-lamp examination. IOP measurements were retrospectively retrieved from the electronic patient database. If a patient received surgical corneal treatment (e.g. a corneal transplant), the last IOP before the surgery was documented, but IOP measurements after the surgery were excluded. Genomic DNA was extracted from whole blood or saliva using a Gentra Puregene Blood kit (Qiagen N.V., Venlo, The Netherlands) or Oragene OG-300 saliva kit (DNA Genotek, Ottawa, Canada). Genotyping was performed using UK Biobank Axiom Array (Applied Biosystems). Genotypes were called using Axiom Analysis Suite software. Imputation of markers was performed using the TOPMed reference panel. SNPs with a SNP call-rate <0.95 or minor allele frequency <0.01 were excluded.

### Genetic Risk Scores

We developed GRS for OAG, CCT, corneal hysteresis (CH), and FECD. We included CH as we hypothesized that this variable might capture more of the cornea's potential for biasing an IOP measurement than CCT. We calculated the GRS for these variables by extracting the significant SNPs and corresponding betas from the largest GWAS for that variable (see Fig. 1).^20^^–^^22^ We then multiplied these betas with the dosage values of the corresponding SNP, and the sum of these multiplications was considered the GRS for any given study participant. The CCT-GRS was validated on the RS data. The FECD-GRS was validated on the FECD cohort. As CH was unavailable in both the RS and the FECD cohorts, we tested the effect of the CH-GRS on CCT. Generally, a thicker cornea becomes more resistant to pressure (assuming no underlying pathology such as corneal edema). We therefore hypothesized that an increased CH-GRS would be associated with increased CCT. For the two-sample MR, the same genetic variants were used for the instrumental exposure variables. The effect of these instrumental variables on OAG was analyzed using OAG summary statistics from a GWAS with an independent sample, namely FinnGen release 12.^23^

### Statistical Analyses

Differences in baseline characteristics between participants with and without OAG were analyzed using independent *t*-tests for continuous variables and chi-square tests (or Fisher's exact tests where applicable) for categorical variables. Overlap between the GRS was tested by estimating the correlation in a univariate linear regression model, and by plotting the linkage disequilibrium (LD) matrices of the variants. Variants with an R^2^ > 0.1 were considered to be in LD, and variants with an R^2^ > 0.5 were considered to be in high LD. For the classical association analyses and one-sample MR, we used binary logistic regression for the OAG outcome, and linear regression models for all other outcomes. Age, sex, and follow-up time were the only other included covariates. We also tested the association of each of the CCT-GRS SNPs with OAG, these analyses were corrected for multiple-testing using the false discovery rate (FDR) according to Benjamini-Hochberg. For analyses of the RS, any participants with a medical history of corneal pathology (i.e. FECD, corneal surgeries, or trauma) or corneal refractive surgeries were excluded. To evaluate the potential confounding effect of topical prostaglandin analogue usage, we repeated the analyses after excluding participants who had used topical prostaglandin analogues before or at the time of CCT measurement. As a secondary analysis, we also performed a longitudinal analysis for the observational and one-sample MR analyses. For this purpose, we used Cox proportional hazard models, adjusted for age and sex, as well as adjusted for age, sex, and IOP, to test for IOP independence. To evaluate nonlinear effects, we fitted the regression models for CCT and CCT-GRS with natural cubic splines, and tested for improvement compared to the linear model using a likelihood ratio test. We also stratified the CCT and the GRSs by quartile using the first quartile as the reference category. Comparisons with the FECD cohort were made with controls from the RS, matched with a 1:4 case:control ratio on age and sex. Comparison of IOP between FECD cases and RS controls was additionally adjusted for the IOP measuring device. Mediation analyses were performed using a bootstrap approach (1000 simulations), mediation was considered present when the average causal mediation effect was significant.^24^ Two-sample MR was performed using inverse variance weighted (IVW), weighted median, simple mode, weighted mode, and MR Egger tests. Sensitivity analyses were performed using the MR Egger intercept to test for pleiotropy, and the heterogeneity Q to test for heterogeneity. All significance tests were 2-sided and a *P* value < 0.05 was considered statistically significant. Analyses were conducted with SPSS version 28.0.1.0 for Windows (IBM Inc., Chicago, IL, USA), and R software version 4.0.3 and R software version 4.2.3 (R Foundation for Statistical Computing) using the tidyverse, forcats, remotes, mediation, TwoSampleMR, LDlinkR, and MRInstruments packages.

## Results

A total of 303 participants with OAG and 10,598 without OAG were included from the RS (Table 1). Participants with OAG were older and more often male. As expected, participants with OAG had a higher IOP, larger vCDR, thinner mRNFL, and thinner mGCC (see Table 1). In total, 162 (1.2%) participants used or had used a topical prostaglandin analogue. There were 753 patients in the FECD cohort (Supplementary Table S1). In both the RS and FECD cohorts, the majority of participants were of European ancestry (99.8% and 92.7%, respectively).

**Table 1.** Baseline Characteristics and Univariate Analyses of the Rotterdam Study Population Without and With OAG

Trait	No OAG (*N* = 10,598)	OAG (*N* = 303)	*P* Value
Age, y	64.9 ± 9.6	68.3 ± 8.4	<0.001
Sex (female, *n*, %)	6147 (58)	146 (48)	<0.001
IOP (mm Hg)^*^	14.3 ± 3.8	19.1 ± 7.3	<0.001
vCDR (median, IQR)	0.3 (0.2 – 0.4)	0.6 (0.3 – 0.8)	<0.001
mRNFL, µm	25.4 ± 5.9	18.3 ± 5.7	<0.001
mGCC, µm	66.2 ± 7.3	55.3 ± 7.5	<0.001
Axial length, mm	23.6 ± 1.4	23.7 ± 2.3	0.660
CCT, µm	548.6 ± 34.0	534.4 ± 35.5	<0.001
CCT-GRS	0.0 ± 1.0	0.0 ± 1.1	0.561
CH-GRS	0.0 ± 1.0	−0.1 ± 1.0	0.102
FECD-GRS	0.0 ± 1.0	0.0 ± 1.0	0.428

CCT, central corneal thickness; CH, corneal hysteresis; FECD, Fuchs endothelial corneal dystrophy; GRS, genetic risk score; IOP, intraocular pressure; IQR, interquartile range; mRNFL, macular retinal nerve fiber layer; mGCC, macular ganglion cell complex; OAG, open-angle glaucoma; vCDR, vertical cup-disc ratio.

Presented as mean ± standard deviation, unless stated otherwise. All genetic risk scores were standardized and therefore expressed in standard deviations.

* Adjusted for IOP-lowering medications (IOP divided by 0.7) and IOP-lowering surgery (IOP set to 30 mm Hg if IOP was below 30 mm Hg prior to adjustment).

### Validation of Genetic Risk Scores

One standard deviation (SD) increase in CCT-GRS was associated with an increase (95% confidence interval [CI]; *P* value) of 10.93 (95% CI = 10.00–11.86, *P* value = 9.22*10^−122^) µm in CCT, and explained 10.3% of the total variance in CCT. The association of the CH-GRS with CCT was not as strong, but still highly significant (*P* = 2.68*10^−52^). Both the CH-GRS and CCT-GRS were strongly associated with measured IOP (*P* = 2.06*10^−9^ and *P* = 1.93*10^−8^, respectively), although only a modest amount of the variance in IOP was explained (R^2^ = 1.1% and R^2^ = 0.8%, respectively). The CCT-GRS was significantly correlated with the CH-GRS (*P* < 0.001), with an R^2^ of 27.3%. A total of 103 out of 234 (44.0%) variants included in the CCT-GRS were in LD (R^2^ > 0.1) with variants from the CH-GRS, of which 78 (33.3%) were in high LD (R^2^ > 0.5). Finally, the odds ratio (OR) for FECD (95% CI, *P* value) was 2.37 (95% CI = 2.13– 2.64, *P* value = 1.09*10^−55^) in the FECD cohort. The area under the curve (AUC; 95% CI) on FECD status was 0.94 (95% CI = 0.94–0.95) for the FECD-GRS.

### Association Analyses

**Figure 2.**
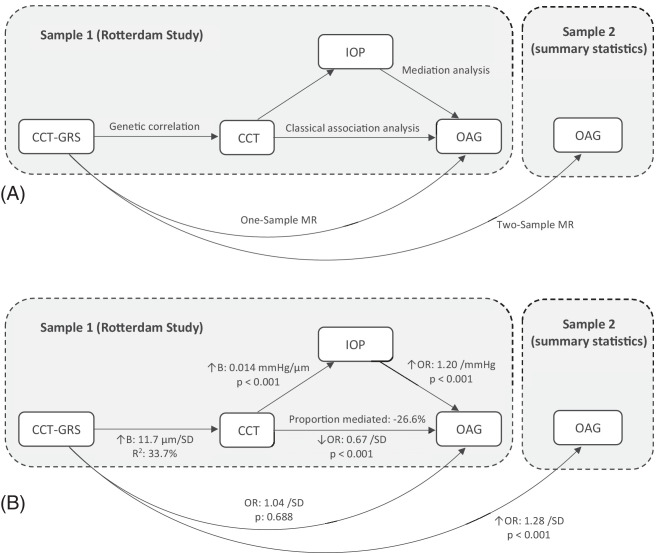
Directed acyclic graphs summarizing the study design (**A**) and results (**B**) for the relation between CCT and OAG. B, beta; CCT, central corneal thickness; GRS, genetic risk score; IOP, intraocular pressure; MR, Mendelian randomization; OAG, open-angle glaucoma; OR, odds ratio; SD, standard deviation.

We found a protective OR (95% CI) of 0.67 (95% CI = 0.56–0.81) for OAG for each SD increase in CCT (*P* < 0.001; Table 2). For this association, there was a statistically significant inverse mediation effect of IOP (*P* < 0.001), with an estimated proportion mediated (95% CI) of −26.6% (95% CI = −67.6% to −15.4%; Fig. 2). Exclusion of participants who had used prostaglandin analogues attenuated the effect of CCT on OAG, but it remained statistically significant (OR [95% CI];OR = 0.79, 95% CI = 0.63–0.98, *P* = 0.034; Supplementary Table S2). Specifically in the thinnest 25% of CCT (i.e. quartile 1), we observed a similar OR (95% CI) of 0.60 (95% CI = 0.46–0.88) for each SD further decrease in CCT. However, the estimated mediation effect of IOP was notably smaller (0.7%) and no longer statistically significant (*P* = 0.64), most likely because CCT did not correlate with IOP in this subgroup (*P* = 0.94). There was no statistical evidence for a nonlinear effect of either CCT (*P* spline = 0.507) or the CCT-GRS (*P* spline = 0.521) on OAG (Supplementary Fig. S1). In the longitudinal analysis, the risk for OAG was significantly reduced with an hazard ratio (HR; 95% CI) of 0.70 (95% CI = 0.58–0.85) for each SD increase in CCT (*P* < 0.001; Supplementary Table S3) without inclusion of IOP in the model, which equals an HR (95% CI) of 1.50 (95% CI = 1.20–1.88) per 40 µm thinning in CCT. Inclusion of IOP slightly magnified this effect, with an HR (95% CI) of 0.65 (95% CI = 0.53–0.79) per SD increase, or 1.65 (95% CI = 1.32–2.07) per 40 µm thinning in CCT. A SD increase in CCT was associated with an average increase (95% CI) of 0.50 (95% CI = 0.39–0.60) mm Hg in measured IOP (*P* < 0.001; see Table 2, Supplementary Fig. S2). For 191 (1.8%) participants, the IOP was imputed due to the use of IOP-lowering medications or previous IOP-lowering surgery. Exclusion of these participants did not significantly alter the results, with an average increase (95% CI) of 0.48 (95% CI = 0.36–0.58) mm Hg per SD increase in CCT. Participants with a thicker CCT had, on average, a smaller vCDR, with a mean difference (95% CI) of −0.009 (95% CI = −0.014 to −0.005) for each SD increase in CCT (*P* < 0.001; see Table 2, Supplementary Fig. S3). We observed no significant association of CCT with either mRNFL or mGCC, nor for the CCT-GRS with mRNFL or mGCC (see Table 2, Supplementary Figs. S4, S5). Participants within the highest quartile of CCT had an OR (95% CI) of 0.44 (95% CI = 0.25–0.79) of OAG compared to the lowest quartile (*P* = 0.006; Supplementary Table S4). In the FECD cohort, patients with FECD had a mean adjusted IOP difference (95% CI) of −1.11 (95% CI = −1.51 to −0.72) mm Hg compared with matched RS controls.

**Table 2.** The Association of Corneal Parameters With OAG and OAG Endophenotypes in the Rotterdam Study

	OR (95% CI)	*P* Value
OAG		
CCT	0.67 (0.56 to 0.81)	<0.001
CCT-GRS	1.04 (0.86 to 1.25)	0.688
CH-GRS	0.97 (0.81 to 1.17)	0.778
FECD-GRS	0.99 (0.83 to 1.20)	0.946
	**Beta (95% CI)**	***P* Value**

IOP^*^
CCT	0.50 (0.39 to 0.60)	<0.001
CCT-GRS	0.18 (0.08 to 0.28)	<0.001
CH-GRS	0.24 (0.14 to 0.35)	<0.001
FECD-GRS	−0.16 (−0.27 to −0.06)	0.002
vCDR		
CCT	−0.009 (−0.014 to −0.005)	<0.001
CCT-GRS	0.003 (−0.001 to 0.007)	0.113
CH-GRS	−0.001 (−0.005 to 0.003)	0.562
FECD-GRS	0.003 (0.000 to 0.007)	0.082
mRNFL		
CCT	0.07 (−0.11 to 0.26)	0.436
CCT-GRS	0.00 (−0.17 to 0.18)	0.980
CH-GRS	−0.01 (−0.18 to 0.17)	0.947
FECD-GRS	0.12 (−0.06 to 0.30)	0.178
mGCC		
CCT	−0.06 (−0.27 to 0.17)	0.625
CCT-GRS	−0.11 (−0.31 to 0.10)	0.318
CH-GRS	−0.11 (−0.32 to 0.10)	0.310
FECD-GRS	−0.12 (−0.33 to 0.09)	0.245

CCT, central corneal thickness; CH, corneal hysteresis; FECD, Fuchs endothelial corneal dystrophy; GRS, genetic risk score; IOP, intraocular pressure; mGCC, macular ganglion cell complex; mRNFL, macular retinal nerve fiber layer; OAG, open-angle glaucoma; OR, odds ratio; vCDR, vertical cup-disc ratio.

Presented as effect estimates per standard deviation increase in corneal parameter. All associations were corrected for age, sex, and follow-up time.

* Adjusted for IOP-lowering medications (IOP divided by 0.7) and IOP-lowering surgery (IOP set to 30 mm Hg if IOP was below 30 mm Hg prior to adjustment).

### One-Sample Mendelian Randomization

We found no association between the CCT-GRS as a continuous variable and OAG (*P* = 0.688; see Table 2), neither after exclusion of participants who used prostaglandin analogues (*P* = 0.335; see Supplementary Table S2), nor in the longitudinal analysis (*P* = 0.964; see Supplementary Table S3), nor when stratified by quartiles (*P* trend = 0.961; Supplementary Table S4). None of the individual SNPs in the CCT-GRS were associated with OAG after correction for multiple testing, however 10 SNPs were nominally significant (*P* < 0.05 uncorrected for multiple testing; Supplementary Table S5). The CCT-GRS was not associated with vCDR (*P* = 0.113), mRNFL (*P* = 0.980), or mGCC (*P* = 0.318; see Table 2). However, the CCT-GRS was associated with 0.18 (95% CI = 0.08–0.28) mm Hg increase in measured IOP per SD increase CCT-GRS (*P* < 0.001). Similarly, we found no association between the CH-GRS and OAG (*P* = 0.778), nor vCDR (*P* = 0.562), mRNFL (*P* = 0.947), or mGCC (*P* = 0.310), but an SD increase in the CH-GRS was associated with an increase (95% CI) of 0.24 (95% CI = 0.14–0.35) mm Hg in IOP (see Table 2). We found no significant association between the FECD-GRS and OAG (*P* = 0.946). The FECD-GRS was, however, associated with a reduced IOP (Beta ,95% CI = −0.16, 95% CI = −0.27 to −0.06 mm Hg per SD). This association sustained after correcting for measured CCT in the model (Beta, 95% CI = −0.18, 95% CI = −0.28 to −0.08 mm Hg per SD, *P* < 0.001).

### Two-Sample Mendelian Randomization

Increasing CCT was associated with elevated OAG risk according to the IVW analysis method, with an OR for OAG (95% CI) of 1.28 (95% CI = 1.20–1.36; Table 3) per SD increase in CCT. The effect was consistent across all other analysis methods. The association between CH and OAG showed similar, but less consistent results (see Table 3). The MR Egger, weighted median, and weighted mode methods showed a similar significant association of CH with OAG risk, but this effect was not replicated with the IVW and simple mode (see Table 3). We found statistically significant evidence for heterogeneity in all two-sample MR analyses (Supplementary Table S6). However, there was no evidence for directional pleiotropy as there was no statistically significant deviation from zero for the MR Egger intercept analyses. Finally, FECD was associated with reduced OAG risk with an OR (95% CI) of 0.92 (95% CI = 0.87–0.98) according to the IVW analysis method. The direction of effect was consistent for all analysis methods, but not statistically significant for the simple mode analysis.

**Table 3.** Two-Sample Mendelian Randomization of Corneal Traits on OAG

Exposure Trait	MR Method	OR (95% CI)	*P* Value
**CCT**	MR Egger	1.23 (1.06 to 1.42)	5.29 × 10^−3^
	Weighted median	1.22 (1.15 to 1.31)	7.60 × 10^−10^
	IVW	1.28 (1.20 to 1.36)	1.88 × 10^−14^
	Simple mode	1.24 (1.03 to 1.49)	2.41 × 10^−2^
	Weighted mode	1.22 (1.09 to 1.37)	7.91 × 10^−4^
**CH**	MR Egger	1.37 (1.05 to 1.80)	2.35 × 10^−2^
	Weighted median	1.30 (1.18 to 1.45)	6.11 × 10^−7^
	IVW	1.08 (0.95 to 1.22)	2.50 × 10^−1^
	Simple mode	1.26 (0.97 to 1.63)	8.00 × 10^−2^
	Weighted mode	1.34 (1.16 to 1.56)	1.59 × 10^−4^
**FECD**	MR Egger	0.87 (0.80 to 0.95)	9.88 × 10^−3^
	Weighted median	0.89 (0.84 to 0.94)	2.63 × 10^−5^
	IVW	0.92 (0.87 to 0.98)	4.80 × 10^−3^
	Simple mode	0.97 (0.86 to 1.11)	0.710
	Weighted mode	0.87 (0.82 to 0.91)	4.66 × 10^−4^

CCT, central corneal thickness; CH, corneal hysteresis; FECD, Fuchs endothelial corneal dystrophy; IOP, intraocular pressure; IVW, inverse variance weighted; MR, Mendelian randomization; OAG, open-angle glaucoma; OR, odds-ratio.

Presented as effect estimates per standard deviation increase in the instrumental variable of the corneal parameter.

## Discussion

### Summary of Findings

In this prospective population-based cohort and MR study, we showed that increased CCT is associated with a reduced risk of OAG and a smaller vCDR, replicating previously reported clinical findings in a population-based cohort.^3^^–^^5^ In one-sample MR analyses, we found no association between a CCT-GRS and OAG. In contrast, in two-sample MR analyses, we found a slightly elevated risk for OAG with increasing CCT. FECD was associated with a lower measured IOP and only associated with reduced risk for OAG in the two-sample MR analyses.

Since the early findings of the Ocular Hypertension Treatment Study, the relation between CCT and OAG has been debated.^3^ In that seminal study, Gordon et al. found an HR (95% CI) of 1.88 (95% CI = 1.55–2.29) per 40 µm thinning of the CCT without adjusting for IOP, and a HR (95% CI) of 1.71 (95% CI = 1.40–2.09) after adjusting for IOP.^3^ Since then, both the Los Angeles Latino Eye Study and the Early Manifest Glaucoma Trial reported similar trends.^4^^,^^5^ Neither study could reproduce a significant association in the unadjusted analyses, but when IOP was included in the model, both reported a significantly increased risk of OAG with an OR (95% CI) of 1.30 (95% CI = 1.00–1.70) and HR (95% CI) of 1.25 (95% CI = 1.01–1.55) per 40 µm thinning of the CCT, respectively.^4^^,^^5^ We found an HR (95% CI) of 1.50 (95% CI = 1.20–1.88) per 40 µm CCT thinning in the analyses unadjusted for IOP. Inclusion of IOP did slightly magnify this effect with an HR (95% CI) of 1.65 (95% CI = 1.32–2.07) per 40 µm thinning of CCT. However, in contrast to our classical observational results, the CCT-GRS was not associated with OAG (*P* = 0.944) or any OAG endophenotypes (except for IOP). Additionally, with two-sample MR we found a moderately elevated OAG risk with increasing CCT. Given the strong and replicated association between CCT and OAG, and the complete lack of any effect, or even a moderate inverse trend, from our MR analyses using the same cohort, confounding bias appears to be highly likely. We propose three most likely hypotheses to explain this observation. First, this effect could be observed due to differences in referral and treatment patterns between patients with high or low CCT. According to the established guidelines, opticians refer patients with a higher measured IOP to an ophthalmologist for OAG screening. It seems likely that patients with higher CCT are, due to artificially inflated measured IOP, referred more swiftly and treated before visual field loss has occurred. Conversely, patients with a low CCT might be not be referred or referred later, at which point glaucomatous visual field loss could already have occurred. In a population-based study, we would find a relation between low CCT and higher OAG risk, despite no direct causal effect of CCT on OAG, no selection criteria for IOP, and an IOP-independent definition of OAG. Although this example is somewhat specific to Europe, visual field-based screening for OAG is currently not cost-effective in high-income countries.^25^ High-income countries therefore currently still use IOP as a referral indicator for OAG, and are exposed to the same referral biases due to the effect of CCT. Second, the use of IOP-lowering medications, in particular prostaglandin analogues, could introduce a confounding bias. Prostaglandin analogues have been shown to reduce CCT, and a large portion (85.5%) of our OAG participants had used or were using a prostaglandin analogue.^26^ However, we also found a significant effect of CCT on OAG when excluding all participants that had used a prostaglandin analogue, although the effect was attenuated. Third, there could be other confounding factors influencing the association between CCT and OAG. Some studies have suggested that CCT could be a biomarker for more general biomechanical properties of the lamina cribrosa and/or other tissues in the eye.^27^ Lower CCT could be correlated with diet, life-style, or other environmental risks that influence both CCT and OAG, although the current literature on this topic is still limited.^28^ Regardless of the presence of a true biological effect, it is clear that CCT is strongly associated with OAG through some direct or indirect effect, and should where possible be included in predictive modeling of OAG risk.

None of the individual SNPs in the CCT-GRS were associated with OAG after correction for multiple testing, however 10 SNPs were nominally significant (see Supplementary Table S5). Interestingly, rs11264763 has previously been associated with reduced retinal thickness.^29^ This is an intron variant in the *JTB* gene, of which overexpression induces swelling of the mitochondria and reduces mitochondrial membrane potential. Increasing evidence suggests the presence of mitochondrial dysfunction in OAG.^30^ Additionally, rs840461 is linked to the *LOX* gene, which codes for collagen chain trimerization and elastic fiber formation. Mutations in this gene have been associated with reduced body height, aortic aneurysms, and dissections.^31^^–^^33^ Similarly, rs12913547 is linked to the *SMAD3* gene, which is a transcription modulator that has also been associated with aortic aneurysms and Loeys-Dietz syndrome (a syndrome characterized by increased arterial aneurysms and tortuosity, as well as osteoarthritis).^34^^,^^35^ Although our MR analyses suggest a direct causal effect of CCT on OAG is unlikely, given the role of these three SNPs in either mitochondrial or connective tissue pathways, and their (nominal) association with both CCT and OAG, a shared genetic effect on both corneal and lamina cribrosa stability appears plausible.

The relation between FECD and OAG is less obvious. The reduced IOP in participants with increased FECD-GRS could be explained by the reduction in corneal rigidity from corneal edema, resulting in lower measured IOP, without necessarily any change in the true IOP. However, CUG-repeat RNA foci, a molecular hallmark of FECD in cases that harbor CTG18.1 expansions (≥50 repeats), have also been detected in the trabecular meshwork (found in 41% of trabecular meshwork cells).^36^ In fact, they are substantially more prevalent in the trabecular meshwork than corneal stromal keratocytes (11% of its cells) or the corneal epithelium (4% of its cells). Corneal endothelium and trabecular meshwork cells originate from neural crest tissue embryonically, exhibit limited proliferative capacity, and may share a common stem cell niche in the transition zone between the termination of endothelium on Descemet’s membrane (Schwalbe’s line) and endothelium of the trabecular meshwork.^37^ It is therefore plausible that CTG18.1 expansion-mediated FECD might therefore actually affect aqueous outflow and true IOP, not just the IOP measurement. This could also explain the small reduction in OAG risk found in our two-sample MR analyses, although we were not able to replicate these results in our one-sample MR experiments. Further research is needed to more definitively assess this relationship.

### Strengths and Limitations

The primary strength of this study is its holistic approach to causal inference. We examined the association between corneal parameters using classical association, one-sample MR, and two-sample MR analyses, in the same cohorts. This allowed us to get a comprehensive understanding of the effects and likelihood of a true causal effect as opposed to systematic biases. Second, we used data from a prospective population-based cohort, with a large study sample representative of the general population and extensive phenotyping. Third, our definition of OAG is also completely IOP independent, including only those participants with reproducible glaucomatous visual field loss, and therefore not introducing any selection bias. Finally, we utilized summary statics from large meta-GWAS to develop our instrumental variables, which were highly correlated with their respective phenotypes. These instrumental variables were applied to a large independent sample from the FinnGen consortium. Our two-sample MR analyses were therefore highly powered to detect an effect, without risking bias from overlapping samples.

There are also some limitations to consider. First, we did not have any CH or FECD data in our population-based cohort, and no OAG phenotyping data in the FECD cohort. We could therefore not analyze the relation between FECD and OAG as comprehensively as for CCT. Second, although we used a large population-based cohort, due to the relatively small prevalence of OAG, the number of OAG cases was relatively low. Third, the GRS only reflected 20% to 40% of the measurement variability of their respective parameters. Although this is relatively high for an MR study, due to our population-based design and the relatively low prevalence of OAG, we might still have been underpowered to detect an effect of the GRS on OAG. Fourth, for the CDR analyses, we accepted linear CDR as the equivalent of vCDR for those participants in which only linear CDR was available. Although linear CDR has previously been shown in this dataset to be at least equally sensitive for OAG compared to vCDR, this measurement heterogeneity might have added some inaccuracy (i.e. statistical noise).^18^ Finally, due to the strictly observational nature of our study, some residual confounding or predisposing factors may have been missed.

### Conclusions and Moving Forward

We found no evidence for a causal link between CCT and OAG. However, CCT measurements are still valuable for population-based risk stratification. Patients with FECD have a lower IOP than controls. However, IOP measurements could be affected by corneal edema. Future studies should investigate if there is an effect on the true pressure inside the eye in patients with FECD, or if this observed association is solely due to a change in biomechanical properties of the cornea. Based on our results, there is no clear relationship between FECD and OAG.

## Supplementary Material

Supplement 1
