# Supplementary material for: Decoding the Cornea-Glaucoma Association: Evidence From Mendelian Randomization
Source: Invest Ophthalmol Vis Sci. 2025 Jul 8;66(9):22. doi: 10.1167/iovs.66.9.22 (PMC12248988; doi:10.1167/iovs.66.9.22)
Supplement: Supplement 1 [file iovs-66-9-22_s001.pdf]

Supplementary appendix

**Decoding the cornea-glaucoma association: evidence from Mendelian Randomization.**

Contents

**Supplementary Table S1.** Baseline characteristics and univariate analyses of the FECD cohort with 1:4 controls age-sex matched controls from the Rotterdam Study.

**Supplementary Table S2.** The association of corneal parameters with OAG and OAG endophenotypes in the Rotterdam Study**,** excluding any cases that used topical prostaglandin analogues before or at the time of CCT measurement.

**Supplementary Table S3.** Multivariable adjusted Cox proportional hazard models of corneal parameters and incident OAG.

**Supplementary Table S4.** The association of corneal parameters with OAG and with IOP in the Rotterdam Study, for each quartile compared to the first quartile.

**Supplementary Table S5.** Association of individual CCT genetic variants that were nominally significantly associated with OAG (uncorrected p-value < 0.05), adjusted for age and sex.

**Supplementary Table S6.** Sensitivity analyses for two-sample Mendelian Randomization analyses, using the MR Egger intercept to test for pleiotropy, and the heterogeneity Q to test for heterogeneity.

**Supplementary Figure S1.** The probability of OAG with corresponding 95% confidence interval for each standard deviation increase in CCT (A) or CCT-GRS (B), using logistic regression with natural cubic splines.

**Supplementary Figure S2.** The IOP with corresponding 95% confidence interval for each standard deviation increase in CCT (A) or CCT-GRS (B), using linear regression with natural cubic splines.

**Supplementary Figure S3.** The vCDR with corresponding 95% confidence interval for each standard deviation increase in CCT (A) or CCT-GRS (B), using linear regression with natural cubic splines.

**Supplementary Figure S4.** The mRNFL with corresponding 95% confidence interval for each standard deviation increase in CCT (A) or CCT-GRS (B), using linear regression with natural cubic splines.

**Supplementary Figure S5.** The mGCC with corresponding 95% confidence interval for each standard deviation increase in CCT (A) or CCT-GRS (B), using linear regression with natural cubic splines.

**Supplementary Table S1.** Baseline characteristics and univariate analyses of the FECD cohort with 1:4 controls age-sex matched controls from the Rotterdam Study.

| Trait | No FECD (N = 3012) | FECD (N = 753) | p-value |
|---|---|---|---|
| Age (years) | 69.3 ± 8.8 | 69.3 ± 8.8 | 0.993 |
| Sex (female, n, %) | 1256 (41.7) | 314 (41.7) | 1.000 |
| IOP (mmHg)* | 14.0 ± 3.2 | 12.9 ± 4.5 | <0.001 |
| IOP device (n, %) | | | |
| - Goldmann applanation | 2990 (100) | 107 (34.6) | |
| - I-care | 0 (0) | 196 (63.4) | N/A |
| - Non-contact tonometry | 0 (0) | 1 (0.3) | |
| - ORA IOPg | 0 (0) | 5 (1.6) | |
| FECD-GRS | -0.4 ± 0.6 | 1.4 ± 1.1 | <0.001 |

Presented as mean ± standard deviation, unless stated otherwise. All genetic risk scores were standardized.

* = adjusted for IOP-lowering medications (IOP divided by 0.7) and IOP-lowering surgery (IOP set to 30 mmHg if IOP was below 30 mmHg prior to adjustment)

FECD = Fuchs endothelial corneal dystrophy, IOP = intraocular pressure, ORA = ocular response analyzer, IOPg = Goldmann correlated intraocular pressure, OAG = open-angle glaucoma, GRS = genetic risk score

**Supplementary Table S2.** The association of corneal parameters with OAG and OAG endophenotypes in the Rotterdam Study, excluding any cases that used topical prostaglandin analogues before or at the time of CCT measurement.

| **OAG** | **OR (95% CI)** | **p-value** |
|---|---|---|
| CCT | 0.79 (0.63, 0.98) | 0.034 |
| CCT-GRS | 0.94 (0.83, 1.07) | 0.335 |
| CH-GRS | 0.90 (0.79, 1.01) | 0.081 |
| FECD-GRS | 0.92 (0.81, 1.05) | 0.238 |

| **IOP*** | **beta (95% CI)** | **p-value** |
|---|---|---|
| CCT | 0.55 (0.46, 0.65) | < 0.001 |
| CCT-GRS | 0.33 (0.25, 0.40) | < 0.001 |
| CH-GRS | 0.24 (0.16, 0.31) | < 0.001 |
| FECD-GRS | -0.13 (-0.20, -0.05) | <0.001 |

| **vCDR** | **beta (95% CI)** | **p-value** |
|---|---|---|
| CCT | -0.007 (-0.012, -0.003) | 0.001 |
| CCT-GRS | 0.002 (-0.001, 0.006) | 0.172 |
| CH-GRS | -0.001 (-0.004, 0.003) | 0.784 |
| FECD-GRS | 0.003 (-0.001, 0.006) | 0.125 |

| **mRNFL** | **beta (95% CI)** | **p-value** |
|---|---|---|
| CCT | 0.03 (-0.16, 0.21) | 0.792 |
| CCT-GRS | 0.04 (-0.13, 0.22) | 0.622 |
| CH-GRS | -0.03 (-0.21, 0.15) | 0.746 |
| FECD-GRS | 0.13 (-0.04, 0.31) | 0.140 |

| **mGCC** | **beta (95% CI)** | **p-value** |
|---|---|---|
| CCT | -0.13 (-0.35, 0.08) | 0.222 |
| CCT-GRS | -0.34 (-0.54, -0.14) | 0.001 |
| CH-GRS | -0.11 (-0.32, 0.09) | 0.282 |
| FECD-GRS | -0.13 (-0.33, 0.08) | 0.225 |

Presented as effect estimates per standard deviation increase in corneal parameter. All associations were corrected for age, sex, and follow-up time.

* = adjusted for IOP-lowering medications (IOP divided by 0.7) and IOP-lowering surgery (IOP set to 30 mmHg if IOP was below 30 mmHg prior to adjustment).

OAG = open-angle glaucoma, OR = odds-ratio, SD = standard deviation, CCT = central corneal thickness, GRS = genetic risk score, CH = corneal hysteresis, FECD = Fuchs endothelial corneal dystrophy, IOP = intraocular pressure, vCDR = vertical cup-disc ratio, mRNFL = macular retinal nerve fiber layer, mGCC = macular ganglion cell layer

**Supplementary Table S3.** Multivariable adjusted Cox proportional hazard models of corneal parameters and incident OAG.

| **Model 1** | **HR (95% CI)** | **p-value** |
|---|---|---|
| CCT | 0.70 (0.58, 0.85) | < 0.001 |
| CCT-GRS | 0.96 (0.85, 1.09) | 0.964 |
| CH-GRS | 0.93 (0.82, 1.05) | 0.240 |
| FECD-GRS | 0.99 (0.86, 1.12) | 0.819 |

| **Model 2** | **HR (95% CI)** | **p-value** |
|---|---|---|
| CCT | 0.65 (0.53, 0.79) | < 0.001 |
| CCT-GRS | 0.95 (0.83, 1.08) | 0.946 |
| CH-GRS | 0.91 (0.80, 1.03) | 0.134 |
| FECD-GRS | 1.00 (0.88, 1.15) | 0.944 |

Presented as effect estimates per standard deviation increase in corneal parameter.
Model 1: Adjusted for age and sex
Model 2: Adjusted for age, sex, and intraocular pressure
OAG = open-angle glaucoma, HR = hazard ratio, CCT = central corneal thickness, GRS = genetic risk score, CH = corneal hysteresis, FECD = Fuchs endothelial corneal dystrophy

**Supplementary Table S4.** The association of corneal parameters with OAG and with IOP in the Rotterdam Study, for each quartile compared to the first quartile.

| OAG, OR (95% CI) | Q1 | Q2 | Q3 | Q4 | p-trend |
|---|---|---|---|---|---|
| CCT | 1.0 | 0.85 (0.53, 1.35) | 0.56 (0.33, 0.95)* | 0.44 (0.25, 0.79)* | 0.002* |
| - Proportion mediated by IOP† | | 18.22% | 29.39% | 32.54% | |
| CCT-GRS | 1.0 | 0.84 (0.61, 1.17) | 1.02 (0.75, 1.40) | 0.93 (0.68, 1.29) | 0.961 |
| CH-GRS | 1.0 | 0.88 (0.65, 1.21) | 0.72 (0.52, 0.99)* | 0.87 (0.64, 1.19) | 0.937 |
| FECD-GRS | 1.0 | 0.88 (0.64, 1.20) | 0.76 (0.55, 1.05) | 0.79 (0.57, 1.09) | 0.093 |

| IOP†, beta (95% CI) | Q1 | Q2 | Q3 | Q4 | p-trend |
|---|---|---|---|---|---|
| CCT | 0.0 | 0.36 (0.06, 0.65)* | 0.78 (0.48, 1.08)* | 1.35 (1.04, 1.66)* | < 0.001* |
| CCT-GRS | 0.0 | 0.10 (-0.11, 0.31) | 0.34 (0.13, 0.55)* | 0.51 (0.30, 0.72)* | < 0.001* |
| CH-GRS | 0.0 | 0.34 (0.13, 0.54)* | 0.37 (0.15, 0.58)* | 0.61 (0.40, 0.82)* | < 0.001* |
| FECD-GRS | 0.0 | -0.26 (-0.48, -0.04)* | -0.38 (-0.59, -0.16)* | -0.37 (-0.59, -0.16)* | < 0.001* |

Presented as effect estimates for each quartile of the corneal parameter compared to the first quartile on either OAG or IOP, with corresponding 95% confidence intervals. All associations were corrected for age and sex.

* = $p < 0.05$

† = adjusted for IOP-lowering medications (IOP divided by 0.7) and IOP-lowering surgery (IOP set to 30 mmHg if IOP was below 30 mmHg prior to adjustment)

OAG = open-angle glaucoma, OR = odds-ratio, Q = quartile, CCT = central corneal thickness, GRS = genetic risk score, CH = corneal hysteresis, FECD = Fuchs endothelial corneal dystrophy, IOP = intraocular pressure

**Supplementary Table S5.** Association of individual CCT genetic variants that were nominally significantly associated with OAG (p-value uncorrected for multiple-testing <0.05), adjusted for age and sex.

| Chromosome | Position* | ID | Effect allele | OR / SD (95% CI) | p-value | FDR p-value |
|---|---|---|---|---|---|---|
| 1 | 153976229 | rs11264763 | T | 1.27 (1.05, 1.53) | 0.012 | 0.87 |
| 2 | 111667724 | rs62162222 | T | 0.78 (0.61, 0.99) | 0.049 | 0.87 |
| 3 | 98813438 | rs6788689 | A | 0.84 (0.71, 0.99) | 0.036 | 0.87 |
| 4 | 86914463 | rs2705620 | T | 0.80 (0.67, 0.96) | 0.014 | 0.87 |
| 5 | 122083564 | rs840461 | T | 0.82 (0.69, 0.96) | 0.016 | 0.87 |
| 5 | 129745671 | rs6595922 | T | 0.81 (0.68, 0.97) | 0.022 | 0.87 |
| 6 | 113053876 | rs2347727 | A | 1.25 (1.02, 1.54) | 0.032 | 0.87 |
| 15 | 67175169 | rs12913547 | T | 1.28 (1.07, 1.54) | 0.009 | 0.87 |
| 17 | 68796113 | rs112549155 | T | 0.75 (0.57, 0.97) | 0.031 | 0.87 |
| 20 | 41052547 | rs17263588 | T | 0.80 (0.64, 0.99) | 0.045 | 0.87 |

* = Positions according to Genome Reference Consortium Human build 38 (GRCh38)

CCT = central corneal thickness, OAG = open-angle glaucoma, OR = odds-ratio, SD = standard deviation, CI = confidence interval, FDR = false discovery rate

**Supplementary Table S6.** Sensitivity analyses for two-sample Mendelian Randomization experiments, using the MR Egger intercept to test for pleiotropy, and the heterogeneity Q to test for heterogeneity.

| Exposure Trait | Method | Q (df) | p-value |
|---|---|---|---|
| **CCT** | MR Egger heterogeneity | 819.81 (225) | $1.45*10^{-68}$ |
| | IVW heterogeneity | 821.17 (226) | $1.69*10^{-68}$ |
| | | **Egger-intercept** | **p-value** |
| | MR Egger intercept | 0.00302 | 0.542 |
| | | | |
| **CH** | **Method** | **Q (df)** | **p-value** |
| | MR Egger heterogeneity | 1021.65 (199) | $1.09*10^{-110}$ |
| | IVW heterogeneity | 1041.42 (200) | $8.38*10^{-114}$ |
| | | **Egger-intercept** | **p-value** |
| | MR Egger intercept | -0.0116 | 0.051 |
| | | | |
| **FECD** | **Method** | **Q (df)** | **p-value** |
| | MR Egger heterogeneity | 25.60 (10) | $4.31*10^{-3}$ |
| | IVW heterogeneity | 32.76 (11) | $5.75*10^{-4}$ |
| | | **Egger-intercept** | **p-value** |
| | MR Egger intercept | 0.0281 | 0.125 |

A statistically significant result ($p < 0.05$) indicates evidence of heterogeneity or pleiotropy, respectively.
OAG = open-angle glaucoma, IOP = intraocular pressure, CCT = central corneal thickness, MR = Mendelian randomization, IVW = inverse variance weighted , CH = corneal hysteresis, FECD = Fuchs endothelial corneal dystrophy

**Supplementary Figure S1.** The predicted probability of OAG with corresponding 95% confidence interval for each standard deviation increase in CCT (A) or CCT-GRS (B), using logistic regression with natural cubic splines. Adjusted for age and sex. The dotted line represents the background probability of OAG (i.e. the probability of OAG for a participant with an average CCT).

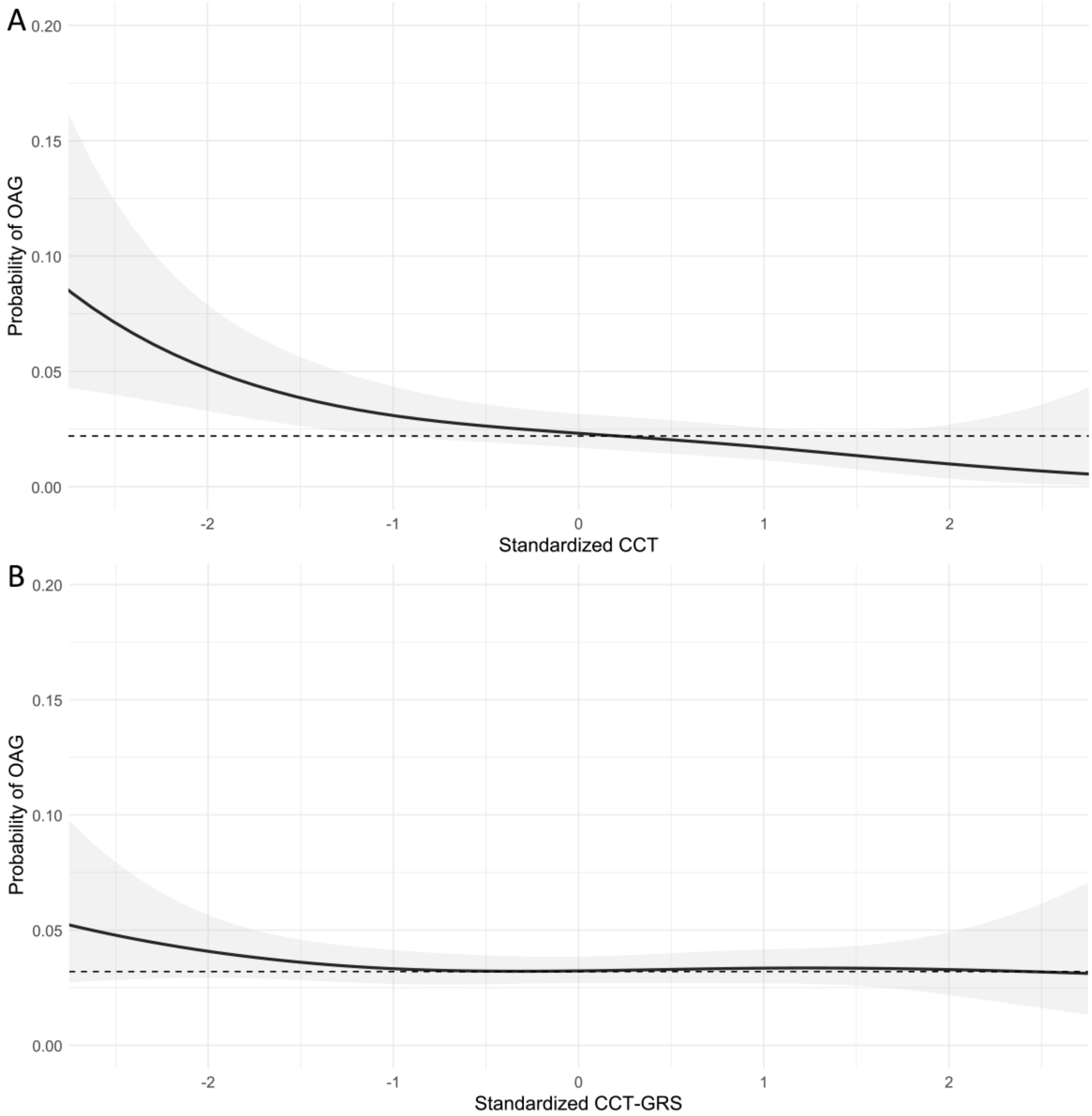


OAG = open-angle glaucoma, CCT = central corneal thickness, GRS = genetic risk score

**Supplementary Figure S2.** The IOP with corresponding 95% confidence interval for each standard deviation increase in CCT (A) or CCT-GRS (B), using linear regression with natural cubic splines. Adjusted for age and sex. The dotted line represents mean IOP.

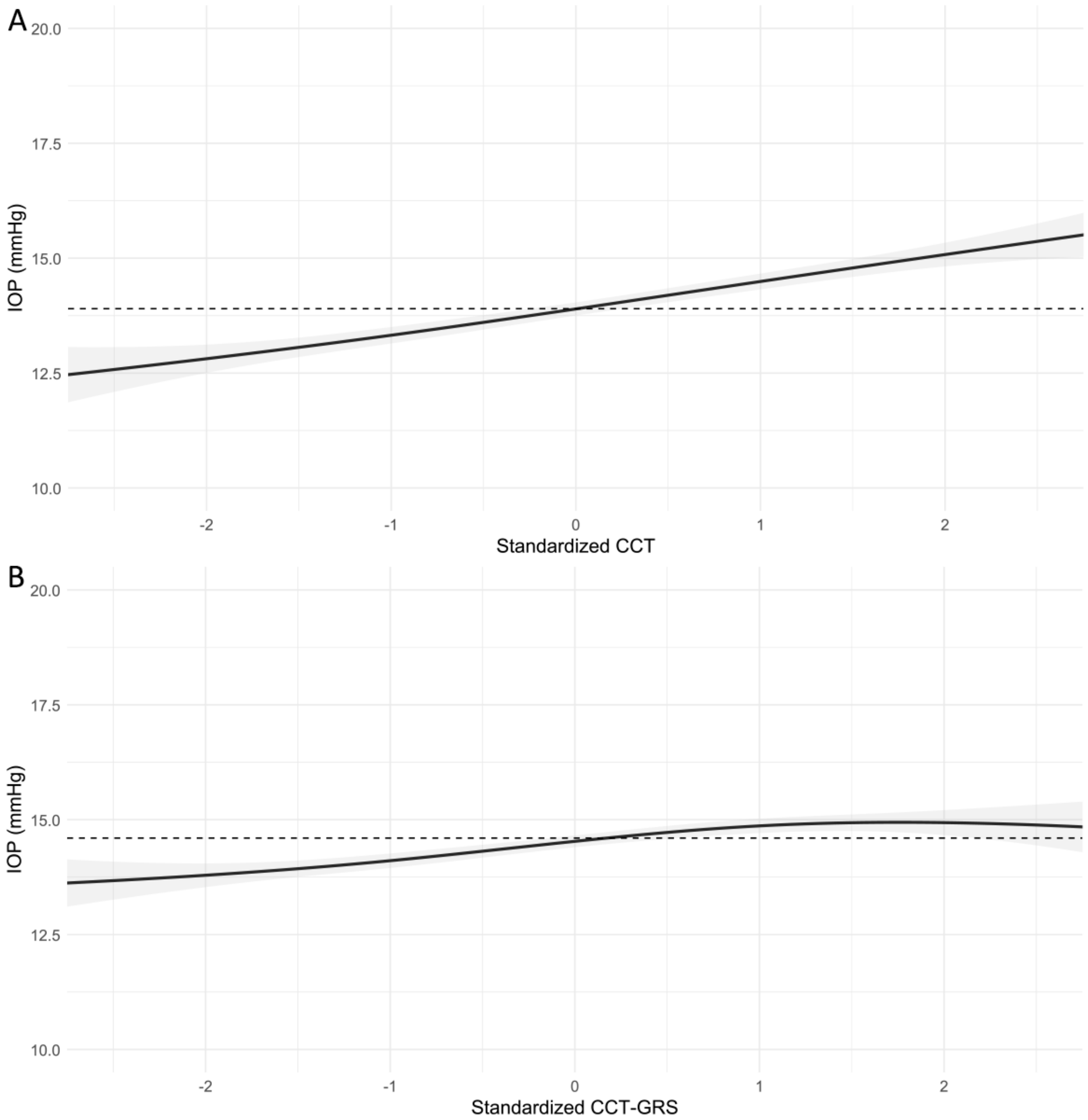


IOP = intraocular pressure, CCT = central corneal thickness, GRS = genetic risk score

**Supplementary Figure S3.** The vCDR with corresponding 95% confidence interval for each standard deviation increase in CCT (A) or CCT-GRS (B), using linear regression with natural cubic splines. Adjusted for age and sex. The dotted line represents mean vCDR.

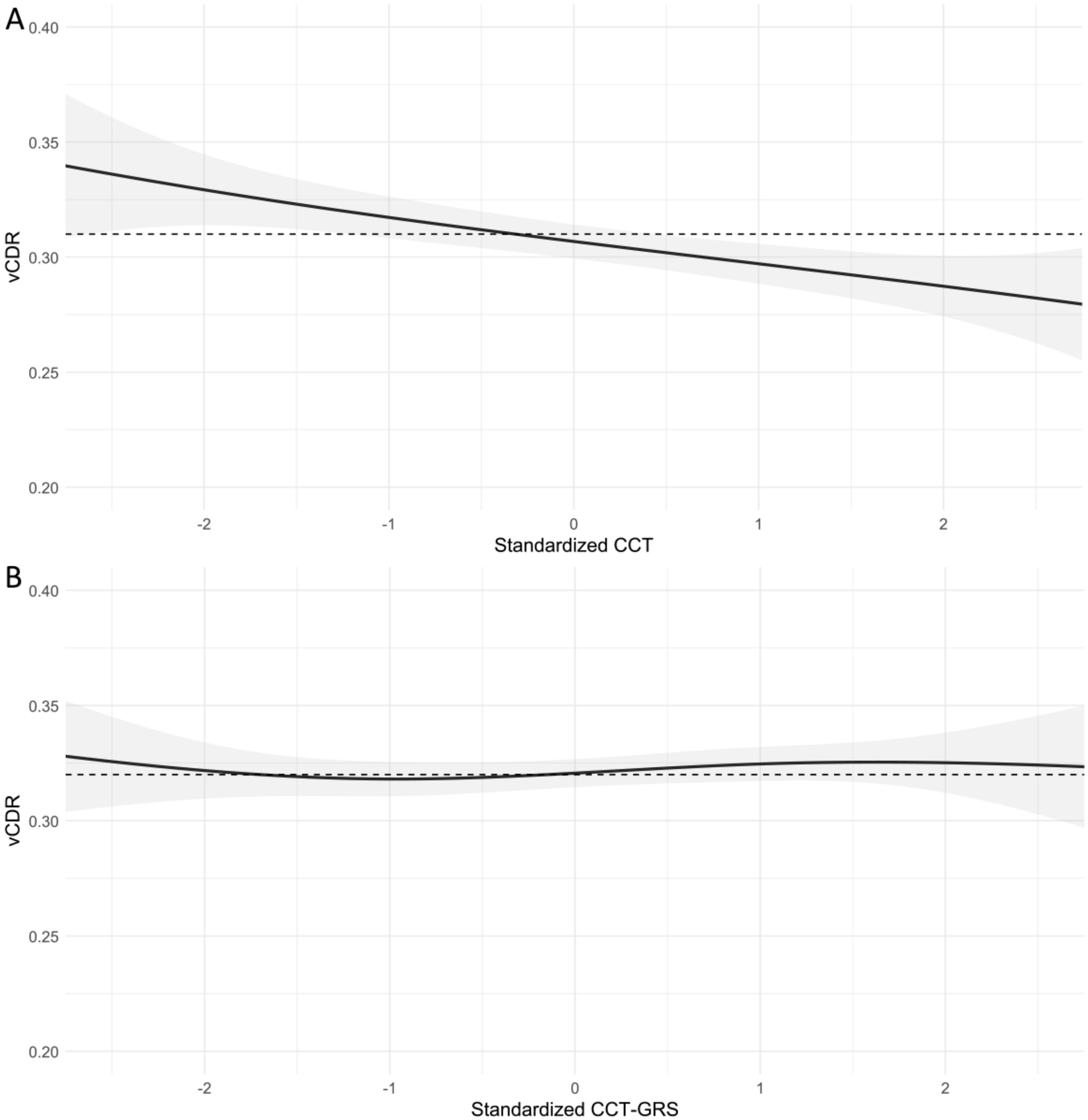


vCDR = vertical cup-disc ratio, CCT = central corneal thickness, GRS = genetic risk score

**Supplementary Figure S4.** The mRNFL with corresponding 95% confidence interval for each standard deviation increase in CCT (A) or CCT-GRS (B), using linear regression with natural cubic splines. Adjusted for age and sex. The dotted line represents mean mRNFL.

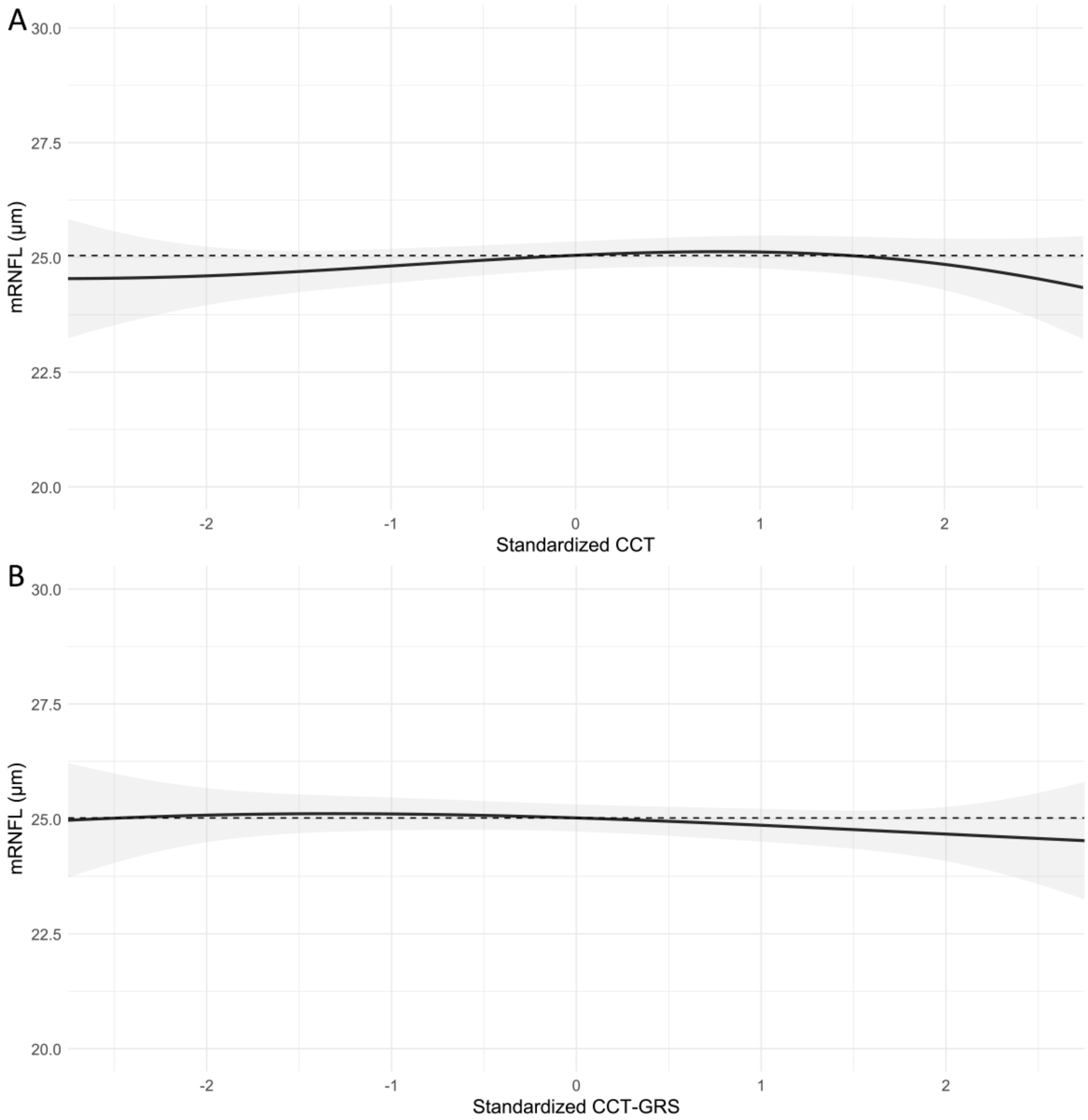


mRNFL = macular retinal nerve fiber layer, CCT = central corneal thickness, GRS = genetic risk score

**Supplementary Figure S5.** The mGCC with corresponding 95% confidence interval for each standard deviation increase in CCT (A) or CCT-GRS (B), using linear regression with natural cubic splines. Adjusted for age and sex. The dotted line represents mean mGCC.

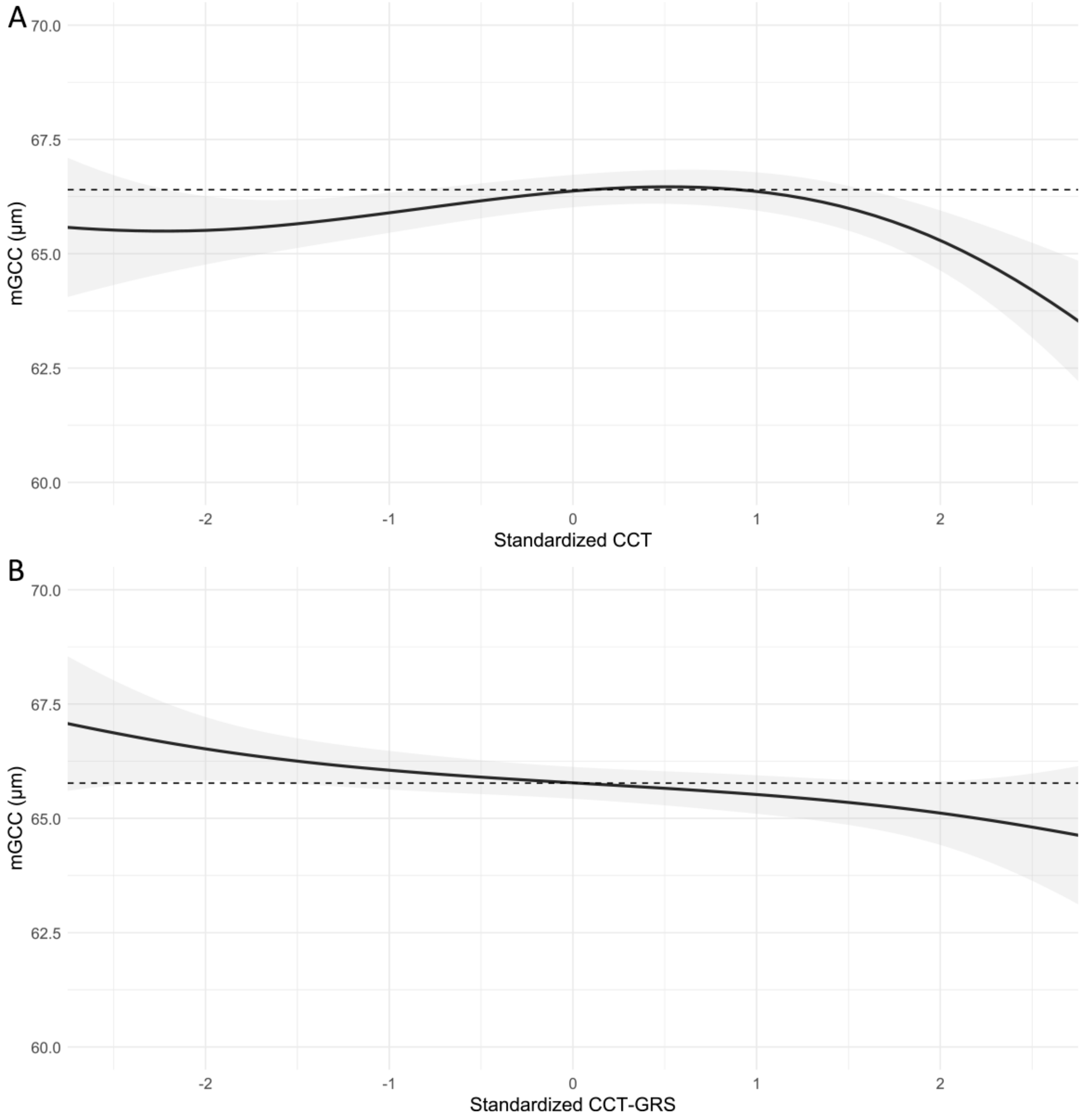


mGCC = macular ganglion cell complex, CCT = central corneal thickness, GRS = genetic risk score
